# Impact of universal screening on MRSA bacteremias in a single acute NHS organisation (2006–12): interrupted time-series analysis

**DOI:** 10.1186/2047-2994-2-2

**Published:** 2013-01-14

**Authors:** Jayanta B Sarma, Bryan Marshall, Victoria Cleeve, David Tate, Tamsin Oswald

**Affiliations:** 1 Northumbria Healthcare NHS Foundation Trust, North Shields, NE29 8NH, UK

## Abstract

**Background:**

In November 2004, a national target was set for the English hospital trusts to reduce the Meticillin-Resistant *Staphylococcus aureus* (MRSA) bacteremia rate by 60% by April 2008 against the number during 2003/04 (baseline year). In our organisation the number of MRSA bacteremias had risen since 2002 and peaked at 75 in 2005/06. A target was set to reduce the number and series of specific and non- specific interventions was introduced including universal MRSA screening. This study analyzes the impact of universal MRSA screening using a quasi-experimental design using routinely gathered data.

**Methods:**

This study used data gathered routinely for clinical governance, quality control, financial management and outbreak monitoring purposes. Interrupted Time Series (ITS) analysis of 15 pre- and 19 post- universal MRSA screening (and decolonisation) quarterly numbers of bacteremias was carried out where Meticillin-Sensitive *Staphylococcus aureus* (MSSA) numbers served as non-equivalent dependent variable (control).

**Results:**

An immediate sharp fall in MRSA bacteremias was observed following the universal MRSA screening (and decolonisation) commenced in Q2, 2007. The number dropped sharply from 23 (Q2, 2007) to 10 (Q3, 2007) for all MRSA bacteremias, and, from 15 (Q2, 2007) to 6 (Q3, 2007) for bacteremias ≥48 hours of hospitalization. The declining trend continued reaching zero in Q2, 2009 and Q4, 2010 for those with ≥48 hours of hospitalization and all bacteremias, respectively. ITS analysis revealed significant impact of universal MRSA screening on all MRSA bacteremias (β_2_ -0.554, *p* 0.000) and those with ≥48 of hospitalization (β_2_ -0.577, *p* 0.001). Impact estimation predicted 17 and 13 bacteremias for all and those with *≥*48 hours hospitalization, respectively in the 19th quarter post-intervention, if the intervention did not occur. The number of MRSA isolates from non-blood culture systemic sources as percentage of admissions also dropped significantly from 3.32% in Q2, 2007 to 1.51% in Q3, 2007 (β_2_ -0.506, *p* 0.000) which is still running low at 0.33% at the end of Q1, 2012. On the other hand, there was no statistically significant impact of universal screening on MSSA bacteremias.

**Conclusions:**

We conclude that of all interventions, the universal MRSA screening (and decolonisation) is the most effective intervention associated with significant and sharp drop in MRSA burden.

## Background

In November 2004, a national target was set for the English hospital trusts to reduce the Meticillin-Resistant *Staphylococcus aureus* (MRSA) bacteremia rate by 60% by April 2008 against the number during 2003/04 (baseline year). In our organisation the number of MRSA bacteremias had risen since 2002 and peaked at 75 in 2005/06. A target was set to reduce the number to 27 bacteremias in the year 2007/08 and an improvement programme was initiated facilitated by Department of Health (DoH) in May 2006. A series of specific- and non-specific interventions was introduced since May 2006. The ‘Clean your hands’ campaign was already ongoing since September 2004. This study reviews the impact of these interventions to reduce the burden of MRSA bacteremias, specifically, the impact of universal MRSA screening using a quasi-experimental (Interrupted Time Series - ITS) design using routinely gathered data in the context of a comprehensive infection control program.

## Methods

The setting is an acute NHS Trust in the north east of England serving a mixed urban/rural predominantly elderly population of approximately 500,000. The Trust has three acute district general and seven community hospitals with 760 acute medical/elderly care beds, and 183 acute surgical beds including orthopedics. The average number of beds per ward is 21 with 82.9% bed occupancy. There is an active infection control team (ICT) with four point five whole time equivalent microbiologists, seven infection control nurses and input from other specialties including pharmacy. The study used no patient identifiable information and is a non-interventional observational study and as such did not require ethical approval.

### Data collection

**Figure 1:**
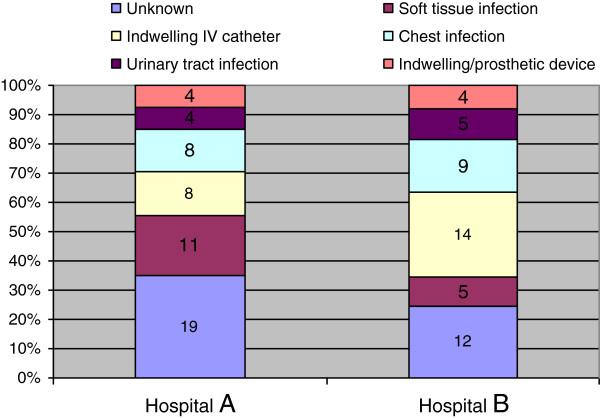
Sources of bacteraemias: September 2006 to March 2007.

This study used data gathered routinely for clinical governance, quality control, financial management and outbreak monitoring purposes. The numbers of monthly bacteremias were routinely recorded for governance purposes. The review of surveillance (Sept 2004 to March 2006) data showed the sources of MRSA bacteremias to be unknown in an unusually large proportion (30%) of cases, and in the rest, three major sources were identified viz., indwelling intra vascular devices, chest infections and soft tissue infections (Figure 1). Following the introduction of Root Cause Analysis (RCA) in May 2006 a number of interventions were made in quick succession as part of the MRSA improvement programme; implementation of these interventions and compliance levels were routinely monitored by audits (Table 1). Universal MRSA Screening and Decolonization was introduced in May, 2007. Before May, 2007 MRSA screening was selective based on certain risk factors including pre-operative patients in elective surgery, emergency orthopedics and trauma surgery, critical care, patients known to be MRSA positive, oncology/chemotherapy inpatients and patients admitted from high-risk settings [1]. The hospitals screened only about 1600 patients per quarter before May, 2007. In May, 2007, the universal screening was introduced in a phased manner to include all adult elective, day case and emergency admissions. This was implemented in advance of the NHS Operating Framework for the NHS in England 2008/09 [2] which required screening for all elective admissions by April 2009 and all emergency admissions no later than 2011.

**Table 1:** Timeline of infection control interventions since Sep 2004

**Year**	**Period**	**Interventions**		**Description (compliance/comments)**
2004	Sep	‘Clean your hands’ campaign [3]		Compliance (against opportunities available) 62-72% at baseline rising to 82% by April 2008. Intense daily monitoring continued with results aggregated and compiled weekly until 95% compliance was achieved at which point frequency was reduced to weekly. In most locations within a few weeks the level of compliance rose to over 95%.
2006	May	Root cause Analysis (RCA)		The information capture tool was given to the clinical teams within hours of the identification of MRSA bacteremia. Once received back by the ICT further in depth investigation (if necessary) was carried out by the Infection Control Nurses (ICN). The clinical teams are updated during the regular visits by Microbiologists or ICNs. More formal feedback occurs during Clinical Governance or Operational board meetings, and also quarterly at Trust Board.
	Jul	Infection Control Nurse Clinical Placement	Junior Doctor’s Induction Video	
	Sep		Support Worker’s Training	
	Nov		The peripheral vascular care (PVC) care plan (High Impact Intervention [4])	Two one day audits in April 2007 and May 2007 showed improvement in usage. Another audit commenced on 22nd October 2007 for 37 consecutive weeks showed the number of cannula *in situ* for >72 hours was only a few and improved compliance to other elements of the care plan viz., (1) removal if no continued clinical indication, (2) use of care plan, (3) daily visual assessment and (4) intact dressing was observed within a few weeks and maintained throughout the period.
	Dec			
2007	May	Universal Screening and decolonization		See result section for full description. 100% compliance both for elective and emergency admissions achieved by beginning April 2009.
		The central vascular care (CVC) care plan		
		Weekly HCAI meeting		A weekly HCAI meeting chaired by the Director of Infection Prevention and Control has been held since May 2007 regularly attended by ICNs, consultant Microbiologists, ward matrons, domestic manager and the director of nursing among others. This group reviews three weekly audits carried out regularly: MRSA screening, PVC care plan and hand hygiene audits in addition to other infection control audits. Another daily meeting for enhanced management of known MRSA positives patients was introduced in March 2008. This group facilitates feedback of results to the relevant staff and ensures that recommended measures are backed by support from high level management in terms of resources.
	Jul	Improved blood culture technique (‘Taking blood cultures: A summary of best practice’ (DoH, June 2007)		The blood culture policy was re-issued which required that indication was recorded in the medical notes and blood culture was authorised by a consultant/senior doctor. A training video was made available on the Trust intranet to demonstrate how to take cultures using aseptic technique. This was also shown at induction for new junior doctors. In June, the number of blood cultures taken fell from the monthly average of 1252 to 778, a 38% reduction, of which 16% were positive compared to 14.5% in the previous five months. The proportion that was skin organisms (e.g., coagulase negative staphylococci, diptheroids and propionibacterium) marginally reduced to 24% from 29%.
		Patient Administration System (PAS) MRSA alert		Patients with previous MRSA history were tagged with an alert code on the PAS to allow for decolonisation to commence within 24 hours of admission in accordance with the MRSA policy.
	Sep	Standardised Intra Venous (IV) cannula site dressing		
		Universal screening compliance audit		See above May, 2007.
		Annual infection control study day and road show		
	Oct	PVC care plan compliance audit		See above November, 2006.
		Web-based Audits Tool		A web based data capture was introduced to audit MRSA screening, peripheral cannula care and hand hygiene standards. This new system enabled the ICT to produce weekly audit figures efficiently at review in the weekly HCAI group meeting, identify the outliers and giving real-time feed back to the ward staff to reinforce corrective measures and best practice in a targeted and timely way.
		ICN job description re-written to make duty and responsibility more explicit		
2008	Jan	NPSA screen saver on all hospital PCs		
		General Practitioner’s education on Infection Control		
		New infection control ward entrance sign		
	Mar	Management of screening positives		Daily meeting for management of screening positives
		MRSA Screening at day 10		Screening of all inpatients at day ten after admission (and then every ten days).
	Apr	Hand hygiene audit		See above Sep 2004 (‘Clean your hands’ campaign)
		Aseptic training for the staff		
	June	Junior doctor’s e-learning on Infection Control		
		Fluoroquinolone restriction		Consumption dropped from average 12 Defined daily Dose (DDD)/100 bed-days in previous 12 months to under 5 from June 2008 with a further drop in consumption to under 2 from June 2010.

Swabs were placed on to MRSA *Select*™ (Bio-Rad) chromogenic agar medium and presumptive pink MRSA colonies were confirmed by a latex slide test. Full biochemical identification and antibiotic susceptibility were carried out by The VITEK® 2 (bioMérieux UK) system. All positive MRSA results were communicated directly to the ICT. Average turnaround time was 48 hours.

**Figure 2:**
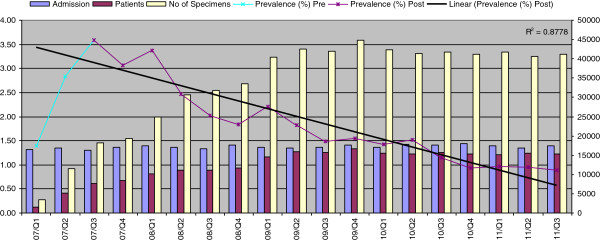
Admissions, patients screened and specimen numbers (secondary axis) vs. on-admission colonisation prevalence (primary axis) from Q1, 2007 to Q3, 2011 (Universal screening began in Q2, 2007).

The number of patients screened increased to 5000 in Q2, 2007 reaching 15000 in Q1, 2009 which has remained stable (Figure 2). A weekly MRSA screening audit commenced in September, 2007 showed near 100% screening compliance achieved by beginning of April, 2009 for all elective and emergency admissions.

The MRSA positive patients were isolated according to the infection control policy and commenced on decolonisation treatment as soon they were identified irrespective of whether facilities were available to isolate. Octenisan® (or 2% Triclosan) body wash once daily and Mupirocin 2% Nasal ointment three times a day to the inside of each nostril for 5 days (and hair washing with Octenisan® on days 2 and 4 of the treatment) were used for decolonisation. A Patient Group Directive (PGD) for prompt prescribing of Octenisan® (or 2% Triclosan) washes by the nursing staff was already in place. Patients are re-screened 48 hours after completing decolonisation if they are still in hospital. In general, up to two complete courses of decolonisation were given during each in-patient stay. Any patient who was still positive for MRSA after a second decolonisation treatment received daily washes using octenisan for the duration of their in-patient stay.

### Analysis

For the interrupted time-series (ITS) analysis, we used segmented linear regression, which divides a time series into pre- and post-intervention segments. The universal screening was introduced in Q2, 2007, therefore, Q3, 2007 was chosen as the intersection between the pre- and post-intervention segments. Interventions that may be short-lived may erroneously report maximal effects if short time series are analyzed; our time series included 19 pre- and 15 post-intervention quarterly data points.

A linear regression model has two parameters: the level and slope. Therefore the difference between the two segments can be quantified by testing the change in these two parameters (Equation 1). A change in level between the pre- and post-intervention segments indicates a step-change, and a change in slope indicates a change in trend.

(1) $$Y_{t}=\beta_{0}+\beta_{1}T+\beta_{2}D+\beta_{3}P$$

Y_t_

is the MRSA rate at month t;

β_0_

estimates the baseline MRSA quarterly rate;

β_1_

estimates the baseline (pre-intervention) linear trend where T is a continuous variable indicating the time in quarter interval at time t from the start of the study period;

β_2_

estimates the level change between pre- and post-intervention, where D = 0 before the intervention, and D = 1 after the intervention;

β_3_

estimates the mean quarterly trend in MRSA post-intervention, compared to the baseline trend, where P is a continuous variable indicating the number of quarters after the start of the intervention at time t and is coded as zero before the intervention.

Three points of interest were calculated: (1) pre-intervention trend (slope); (2) post-intervention change of the baseline level representing an immediate effect; and (3) post-intervention trend (slope) representing a sustained effect of the intervention. A *p*-value of 0.05 was regarded as statistically significant. If coefficient β_1_ was significantly different from 0, the pre-intervention trend was deemed statistically significant. Similarly, if β_2_ and/or β_3_ were significantly different from 0, it was assumed that there had been a significant post-intervention change of baseline level and/or trend.

We included Meticillin-Sensitive *Staphylococcus aureus* (MSSA) numbers for the same period as non-equivalent dependent variable [5] which would be affected by all infection control interventions except the MRSA universal screening. ITS analysis for both MRSA and MSSA series was carried out, Q3, 2007 as the intersection between segments. The null hypothesis was that changes in the positive blood cultures and positive systemic samples other than blood cultures for MRSA and the MSSA were similar.

We have included Durbin-Watson (DW) in the regressions statistics to correct for possible auto-correlation. DW value between 1.5 and 2.5 indicates that the values are independent. All analysis was done using SPSS 19.

## Results

### Patients screened

The number of patients screened almost trebled from 1642 (3,432 specimens) in Q1, 2007 to 5087 (11,518 specimens) in Q2, 2007 then increased gradually over the next quarters to finally stabilize over 15000 (40,000 specimens) by Q1, 2009 (Figure 2). The ratio of patients to specimen numbers was about 1:2 in line with the swabbing of nose (N) and throat (T) only. However, this ratio has increased since Q2, 2008 to just under 1:3 when we introduced inpatient MRSA screening at every 10 days interval. The highest detection of on-admission prevalence of colonized patients was achieved (3.59%) in the Q3, 2007 then sharply reduced to 2%, Q2, 2008), 1%, Q3, 2010) and, finally, dipped down to 0.89% in Q4, 2010 (R^2^ = 0.9), clearly reflecting the efficacy of decolonization and successful eradication (Figure 2).

### Estimation of baseline prevalence of MRSA colonization

The highest rate of MRSA positive systemic samples (infections) other than blood cultures was 6.47% of all admissions in Q1, 2005 which implies a rate of colonization prevalence much higher than 6.47%. What was the on-admission colonization rate could not be extrapolated from inpatient infection burden but in Q1, 2007 the on-admission colonization detection rate was only 1% with risk based targeted screening. The highest on-admission detection rate of 3.59% was achieved in Q3, 2007 after universal screening was commenced (Figure 2). The impact of the universal screening and decolonisation on the MRSA burden was immediate: there was a sharp decline in on-admission colonisation prevalence from a peak at 3.59% in Q3, 2007 to less than 1% by Q4, 2010, while the number of patients screened remained stable at just over 15,000/quarter. These data reflect that detection by targeted screening before the universal screening was by far less efficient.

### Impact on MRSA bacteremias

The target set required a reduction from 67 to 27 bacteremias by April, 2008 equivalent to numbers falling from 5.6 cases to 2.3 cases per month compared to the baseline year. Initially, following the introduction of RCA in May 2006, a tangible reduction in the numbers was achieved on a month by month basis. A care pathway for insertion, management and maintenance of central and peripheral lines was introduced (November, 2006). However, in the subsequent months there was a gradual increase with MRSA bacteremias peaking at 23 cases in Q2, 2007. The universal MRSA screening was introduced in Q2, 2007 and an immediate and drastic reduction in MRSA bacteremias was seen in Q3, 2007; the declining trend continued till Q1, 2012 with only two cases ≥48 h of hospitalization (hospital acquired) occurring in 2011–12.

Visual inspection of data (Excel graphics) revealed an immediate effect of universal screening in terms of sharp decline of MRSA bacteremias as well as on positive systemic (non-blood culture) samples in line with the decline of MRSA on-admission prevalence.

**Figure 3:**
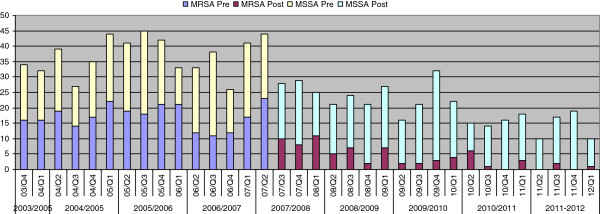
MRSA and MSSA bacteraemias (all) pre- and post-universal screening.

**Figure 4:**
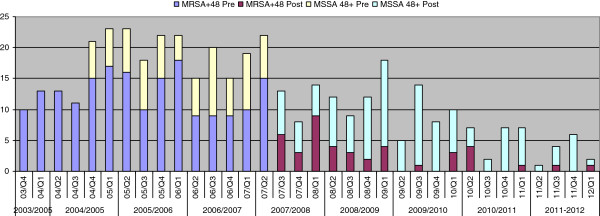
**MRSA and MSSA**^**1 **^**bacteraemias ≥48 h of hospitalization pre- and post-universal screening.**

There was an immediate sharp fall in all MRSA bacteremias from 23, Q2 2007 to 10, Q3, 2007 followed by a continued declining trend reaching zero Q4, 2010 (Figure 3). Predicted regression line showed a slight decreasing trend in the slope before the intervention, an abrupt drop in the rate immediately following the intervention, and a gradually decreasing slope continued after the intervention. The hospital acquired MRSA bacteremias (≥48hours of hospitalization) dropped from 15 in Q2 2007 to 6 in Q3, 2007 followed by a continued declining trend reaching zero in Q2, 2009 (Figure 4). These observations were tested using interrupted time-series (ITS) analysis. The ITS analysis (Table 2) revealed significant post-intervention drop of the baseline level of all MRSA bacteremias, representing an immediate effect, (β_2_ -0.554, *p* 0.000) and the post-intervention declining trend (slope), representing a sustained effect (β_3_ -0.393, *p* 0.048) where pre-intervention coefficient β_1_ was close to 0 (−0.033,) signifying a flat trend. The immediate effect was equally prominent for hospital acquired MRSA bacteremias i.e., ≥48 h of hospitalization (β_2_ -0.577, *p* 0.001) but post-intervention slope was not statistically significant (β_2_ -0.216, *p* 0.298) as the numbers were already very low after the immediate sharp drop.

**Table 2:** Interrupted time-series regression analysis of the MRSA/MSSA bacteremias (total and ≥48 h), MRSA/MSSA systemic (Hospital - % of admissions) and MRSA/MSSA systemic (Community – total numbers)

	**Parameter**	**Unstandardized**	**Standardized**	**t**	***p***	**95% CI**	**D-W**
		**Coefficients - B (Std. Error)**	**Coefficients - β**				
**MRSA bacteremias**	Constant	17.40 (1.64)		10.59	.000	14.04 to 20.75	1.573
	Pre-intervention trend	-.025 (.181)	-.033	-.138	.891	-.39 to .34	
	Post-intervention change	−8.32 (2.072)	-.554	−4.016	**.000**	−12.55 to −4.09	
	Post-intervention trend	-.45 (.221)	-.393	−2.065	**.048**	-.90 to -.005	
**MSSA bacteremias**	Constant	17.36 (2.45)		7.075	.000	12.35 to 22.37	2.068
	Pre-intervention trend	.29 (.270)	.604	1.098	.281	-.255 to .848	
	Post-intervention change	−2.28 (3.095)	-.235	-.737	.467	−8.60 to 4.04	
	Post-intervention trend	-.61 (.330)	-.818	−1.858	.073	−1.28 to.06	
**MRSA ≥48 h bacteremias**	Constant	13.46 (1.38)		9.75	.000	10.64 to 16.28	1.753
	Pre-intervention trend	-.10 (.152)	-.168	-.659	.515	-.41 to .21	
	Post-intervention change	−6.79 (1.741)	-.577	−3.901	**.001**	−10.34 to −3.23	
	Post-intervention trend	-.19 (.185)	-.216	−1.060	.298	-.57 to .18	
**MSSA ≥48 h bacteremias**	Constant	5.96 (1.84)		3.22	.003	2.16 to 9.76	1.901
	Pre-intervention trend	.173 (.272)	.503	.634	.532	-.38 to .73	
	Post-intervention change	1.10 (2.112)	.178	.521	.606	−3.24 to 5.44	
	Post-intervention trend	-.45 (.298)	-.990	−1.524	.140	−1.06 to .15	
**MRSA Systemic (Trust)**	Constant	5.8 (.225)		25.78	.000	5.34 to 6.26	1.599
	Pre-intervention trend	-.17 (.028)	-.830	−6.252	.**000**	-.23 to -.11	
	Post-intervention change	−2.03 (.271)	-.506	−7.493	**.000**	−2.58 to −1.47	
	Post-intervention trend	.10 (.033)	.350	3.269	**.003**	.040 to 17	
**MSSA Systemic (Trust)**	Constant	3.25 (.191)		17.01	.000	2.86 to 3.64	1.421
	Pre-intervention trend	-.05 (.024)	-.892	−2.358	**.026**	-.10 to -.007	
	Post-intervention change	.29 (.230)	.248	1.289	.208	-.17 to .76	
	Post-intervention trend	.134 (.028)	1.477	4.846	**.000**	.07 to .19	
**MRSA Systemic (GP)**	Constant	287 (12.35)		23.25	.000	262 to 313	1.962
	Pre-intervention trend	−2.49 (1.55)	-.285	−1.602	.120	−5.68 to .69	
	Post-intervention change	−66.23 (14.89)	-.402	−4.447	**.000**	−96.73 to −35.72	
	Post-intervention trend	−4.22 (1.78)	-.338	−2.362	**.025**	−7.88 to -.56	
**MSSA Systemic (GP)**	Constant	906 (31.57)		28.71	.000	841 to 971	2.427
	Pre-intervention trend	−7.52 (3.97)	−1.27	−1.891	.069	−15.67 to .62	
	Post-intervention change	11.71 (38.04)	.105	.308	.760	−66.22 to 89.65	
	Post-intervention trend	10.16 (4.56)	1.20	2.224	**.034**	.80 to 19.51	

Impact estimation predicted 17 (all) and 13 (≥48 hours of hospitalization) MRSA bacteremias, respectively in the 19th quarter post-intervention if the intervention did not occur, and, predicted zero bacteremias with intervention which is borne out by the observation.

**Figure 5:**
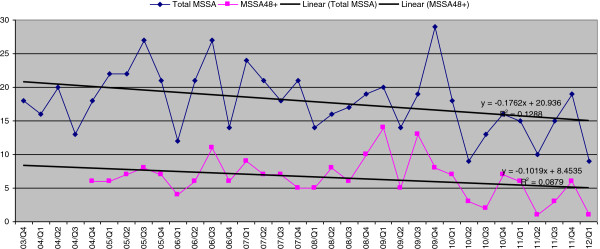
MSSA bacteraemias trend pre- and post-universal screening.

There was no statistically significant impact of universal screening on MSSA bacteremias. During the whole observation period of 34 consecutive quarters the number of MSSA bacteremias, both community onset (R^2^ = 0.13) and ≥48 hours of hospitalization (R^2^ = 0.09) have been declining slowly but not at a statistically significant level (Figure 5, Table 2).

### Impact on systemic Non-blood culture specimens

**Figure 6:**
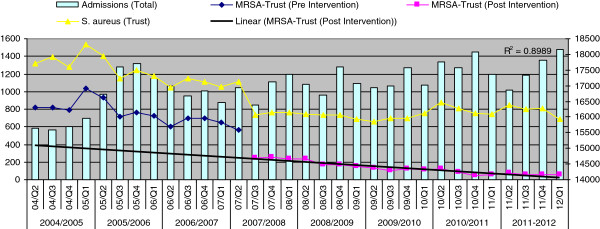
**Non-blood culture systemic MRSA vs. *S. aureus *(primary axis) isolates with number of admissions (secondary axis) between Q4, 2002 to Q1, 2012.**

Similarly, there has been an equally significant impact on the number of MRSA isolates from non-blood culture systemic sources: sterile fluid, tissues, respiratory specimens and swabs, superficial and deep (Figure 6). The number of positive specimens as percentage of admissions dropped from 3.32% in Q2, 2007 to 1.51% in Q3, 2007. The declining trend continued steadily over the following quarters to the lowest 0.25% in Q4, 2010 (R^2^ = 8.9). The ITS analysis revealed a pre-intervention declining trend (β_1_ -0.830, *p* .000) but a significant sharp post-intervention drop representing an immediate effect (β_2_ -0.506, *p* 0.000). However, there was no prominent post-intervention declining trend (β_3_ 0.350, *p* 0.003) as the rate already dropped and was running at a very low level at < 0.3%. Percentage of MRSA of all *S. aureus* isolates (non-blood culture systemic sources) declined from 51% Q2, 2007 (peak was at 67.54% in Q3, 2005) through 34% Q3, 2007 to 8.7% in Q1, 2012.

The immediate decline and post-intervention declining trend were also significant for MRSA non-blood culture systemic specimens from general practitioners (GP): β_2_ -0.402, *p* 0.000; β_3_ -0.338, *p* 0.025 whereas pre-intervention declining trend was not significant (Table 2).

**Figure 7:**
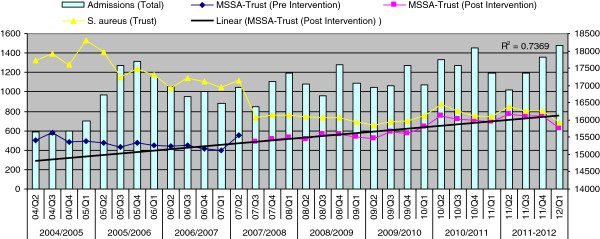
**Non-blood culture systemic MSSA vs. *S. aureus *(primary axis) isolates with number of admissions (secondary axis) between Q4, 2002 to Q,1 2012.**

Of note, however, there was no concomitant reduction in MSSA isolates from non-blood culture systemic sources observed. Instead, there was a slow but statistically significant post-intervention increasing trend in the rate of hospital MSSA (R^2^ = 7.3, Figure 7) confirmed by ITS analysis (β_3_ 1.477, *p* 0.000). The ITS analysis also revealed post-intervention increasing trend in the numbers of non-blood culture systemic MSSA from GP (β_3_ 1.20, *p* 0.034) although the predicted regression line was not significant (R^2^ = 0.06).

### Cost of screening

Each specimen was associated with a consumable cost of £0.62 for a negative screen and, additional, £2.16 for a positive specimen (identification and sensitivity). Estimated cost of screening was £24,800 per quarter plus £1080 based on the current quarterly positive number of about 500. The yearly cost of universal screening was just over £100,000 for laboratory consumables alone. In Q1, 2012 a combined NP Elution Swab (ESwab) in Liquid Amies medium was introduced reducing the number of specimens to half. The Eswab costs £0.45 more compared to the standard Amies swab, so, total consumable cost would be only marginally lower but specimen numbers halved.

### Mupirocin resistance

The resistance to mupirocin (hospital isolates) marginally increased from 1.7% in 2004 to 2.3% in 2011.

## Conclusion

In the era of multi-resistant pathogens, focusing hospital resources on a single antibiotic-resistant pathogen as a sole approach to infection control is criticized as inherently flawed [6]. Questions were also raised regarding the impact of universal screening on MRSA bacteremia rate given the sensitivity of the screening, lack of isolation rooms, the relatively low effectiveness of current decolonization techniques, staffing time and financial resources required vis-à-vis (cost-) effectiveness and the real reduction in risk achieved. Caution is advised for more evidence before it becomes routine considering resource requirements, practical difficulties and consequences [7]. In hospitals with an MRSA on-admission prevalence of < 5% targeted rather than universal screening is advocated [8].

In our study, all interventions were targeted at both MRSA and MSSA except the screening and decolonization but decline in MSSA bacteremias over the 34 consecutive quarters of observation was not statistically significant, which served as a natural control (non-equivalent dependent variable) in the analysis. In contrast, a drastic drop and quarterly declining trend in MRSA bacteremias continued, reaching zero following the introduction of universal screening. In national context, during the first 2 years following setting of the national target in November 2004, there was minimal reduction; however, from September 2006 onwards, rates declined dramatically to reach a reported 57% reduction by April–June 2008 [9-11]. In our organization, dramatic decline in MRSA bacteremias and systemic infections was clearly linked to the introduction of universal screening and decolonization. This is consistent with reports that widespread uptake of decolonization has made the key additional contribution [11,12]. The effect of hand hygiene, identification of patients with MRSA infections or colonization and isolation interventions alone leads only to a gradual reduction in MRSA burden over many years [13]. The Pathfinder project [14], an implementation project to evaluate the impact of universal screening recommended by the NHS QIS HTA modeling [15], also found a temporal association between the initiation of universal screening and a significant reduction in colonization from 5.5% to 3.5% by month 12. However, no significant reduction in the rate of MRSA bacteremias and Surgical Site Infections (SSI) during the study was found. A re-worked model using observed parameters from the Pathfinder study projected that colonization prevalence could reduce over three to five years to low endemic levels (0.5-1.8%) with a chromogenic agar based universal screening strategy. Our study results support this projection.

In one neighbouring NHS Trust only seven additional day case patients in one month period would have been identified as MRSA carriers using the DoH universal screening compared with their targeted approach [16]. However, this Trust had already embarked on an expanded screening programme in three phases and needed to extend the screening only to day cases and elective admissions to comply with mandatory universal screening commenced in April, 2009. It is only to be expected that a successful screening and decolonization programme over a period would eventually lower the burden and a corresponding fall in MRSA detection rate.

In previous studies fluoroquinolone restriction was associated with decline in MRSA infections [17]. In our study MRSA rates dropped during the period when fluoroquinolone were used unrestricted albeit at a low level of average 12 DDD/100 bed-days per month. Our Fluroquinolone consumption more than halved to under 5 DDD/bed-days from June, 2008 which further dropped to under 2 from June, 2010. This low level of consumption may have positively impacted on our sustained low burden of MRSA infections.

Once low on-admission prevalence has been consolidated and sustained, a first line ‘risk assessment’ screening tool to reduce swabbing, laboratory costs and identify the proportion of high risk patients who could be preemptively isolated is a logical way forward. One study found that a simple three question Clinical Risk Assessment (CRA) could perform to a similar level to universal single site swabbing, but with considerably reduced resource implications [15]. The CRA has to be based on the local MRSA epidemiology, infection control practices and vulnerability of the patient population.

The ITS methods used in this study have several advantages over other quasi-experimental studies, because they are less likely to be influenced by certain biases [18-20]. Cyclical effects and underlying increasing or decreasing secular trends may contribute to observed intervention effects. While looking at the effect of specific intervention, the analysis has to take into account the added effect of pre-existing or newly introduced measures. Finally, auto-correlated data means that adjacent data points can be more similar (positive auto-correlation) or dissimilar (negative auto-correlation), leading to under- or over-estimates of effect, respectively. Unlike the classical statistical methods that assume the observed data are independent random variables, time-series analysis takes into account the relationships existing between consecutive observations, a phenomenon known as auto-correlation. However, as with all non-experimental designs, causal inference from ITS designs is limited because it is impossible to rule out alternative explanations for observed changes in the time series.

Our experience with the precipitous decline in the burden of MRSA provides clear evidence of the efficacy of universal screening and decolonization. We achieved a very high level of compliance with screening uptake and decolonization. However, this success has to be considered in the context of the highly empowered weekly HCAI meeting in operation since May, 2007. This group have been ensuring continued compliance to MRSA screening, PVC care plan use and hand hygiene reviewing weekly audits and feeding back to the relevant staff with timely recommendation of appropriate measures backed by the support from the management.

It is noteworthy that our success was in the background of a declining trend in MRSA infections in England [11], the USA [21] and elsewhere during the period. If there is an epidemiological explanation, perhaps, an effect predicated on natural biological trends involving specific MRSA strains is a matter of great interest [11,22,23].

The study reveals one disturbing trend. The rate of non-blood culture systemic MSSA positive samples from the trust since Q2, 2007 is increasing. Also, the MSSA bacteremia trend appears to be decreasing only slowly and not at a significant level. This warrants specific actions to combat the MSSA burden.

## Competing interests

The authors declare that they have no competing interests.

## Authors’ contributions

JBS prepared the manuscript. All authors contributed to the analysis and reviewed the manuscript.

## Authors’ information

BM is Director of Infection Control and Prevention.

JBS, VC, DT and TO are Consultant Microbiologists and Infection Control Doctors.
